# WITHIN TWIN PAIR DIFFERENCES INDICATE GENETIC EFFECTS OF SES INDICATORS ON LONGITUDINAL CHANGES IN PHYSICAL AGING

**DOI:** 10.1093/geroni/igad104.3698

**Published:** 2023-12-21

**Authors:** Deborah Finkel, Malin Ericsson, Margaret Gatz

**Affiliations:** University of Southern California, Los Angeles, California, United States; Karolinka Institutet, Stockholm, Stockholms Lan, Sweden; University of Southern California, Los Angeles, California, United States

## Abstract

Socioeconomic status (SES) predicts change in health status over age, even after accounting for measured confounders such as environmental and biological risk factors. Nonetheless, the source of SES-health associations continues to be heavily debated. Twin studies offer a method for testing causal hypotheses by incorporating within and between twin pair differences in longitudinal latent growth curve models (LGCM) of physical aging on both level of functioning and rate of change with age. Three longitudinal twin studies of aging from the Swedish Twin Registry (N = 2059) included up to 27 years of follow-up on a Functional Aging Index (FAI) consisting of lung function, grip strength, walking speed, and self-report sensory functioning. SES indicators included education, financial strain, and occupation. Pair means (between family effect) and within pair differences (within family effect) were included as covariates of both intercept and slopes in a two-slope LGCM (intercept at age 75); models were corrected for sex and parental SES. LGCM results were compared across full sample, monozygotic twins, and dizygotic twins. For the FAI intercept, MZ within pair effects in all SES variables were strongly attenuated, indicating genetic confounding. For slope 1, only MZ within pair effect for education was attenuated, and no SES indicators were related to rate of change in FAI after age 75. Thus, results suggest shared genetic factors explain the association between education and change in physical aging in early aging processes. In other words, genes that impact educational achievement may also influence health behaviors and physical aging.

